# Celecoxib-Induced Modulation of Colon Cancer CD133 Expression Occurs through AKT Inhibition and Is Monitored by ^89^Zr Immuno-PET

**DOI:** 10.1155/2022/4906934

**Published:** 2022-01-07

**Authors:** Kyung-Ho Jung, Jin Hee Lee, Mina Kim, Eun Ji Lee, Young Seok Cho, Kyung-Han Lee

**Affiliations:** ^1^Department of Nuclear Medicine, Samsung Medical Center, Sungkyunkwan University School of Medicine, Seoul, Republic of Korea; ^2^Samsung Advanced Institute for Health Sciences & Technology, Sungkyunkwan University School of Medicine, Seoul, Republic of Korea

## Abstract

We developed an immuno-PET technique that monitors modulation of tumor CD133 expression, which is required for the success of CD133-targeted therapies. *Methods*. Anti-CD133 antibodies were subjected to sulfhydryl moiety-specific ^89^Zr conjugation. ^89^Zr-CD133 IgG was evaluated for specific activity and radiolabel stability. Colon cancer cells underwent binding assays and Western blotting. Biodistribution and PET studies were performed in mice. *Results*. ^89^Zr-CD133 IgG showed excellent target specificity with 97.2 ± 0.7% blocking of HT29 cell binding by an excess antibody. Intravenous ^89^Zr-CD133 IgG followed biexponential blood clearance and showed CD133-specific uptake in HT29 tumors. ^89^Zr-CD133 IgG PET/CT and biodistribution studies confirmed high HT29 tumor uptake with lower activities in the blood and normal organs. In HT29 cells, celecoxib dose-dependently decreased CD133 expression and ^89^Zr-CD133 IgG binding that reached 19.9 ± 2.1% (*P* < 0.005) and 50.3 ± 10.9% (*P* < 0.001) of baseline levels by 50 *μ*M, respectively. Celecoxib treatment of mice significantly suppressed tumor CD133 expression to 67.5 ± 7.8% of controls (*P* < 0.005) and reduced tumor ^89^Zr-CD133 IgG uptake from 15.5 ± 1.4% at baseline to 12.3 ± 2.0%ID/g (*P* < 0.01). Celecoxib-induced CD133 reduction in HT29 cells and tumors was associated with substantial suppression of AKT activation. There were also reduced HIF-1*α* accumulation and I*κ*B*α*/NF*κ*B phosphorylation. *Conclusion*. ^89^Zr-CD133 IgG PET provides high-contrast tumor imaging and monitors celecoxib treatment-induced modulation of tumor CD133 expression, which was found to occur through AKT inhibition. This technique may thus be useful for screening drugs that can effectively suppress colon cancer stem cells.

## 1. Introduction

Colorectal cancer is a leading cause of morbidity and mortality worldwide, and treatment-resistant cancer stem cells (CSCs) are a major challenge for overcoming this disease [1, 2]. The most widely used biomarker to identify and target colon CSCs is CD133 (prominin 1), a transmembrane protein associated with tumor progression and poor patient outcomes. Cancer cells that express CD133 have the potential for multilineage differentiation and are capable of tumor initiation in vivo [3, 4]. In patients with colon cancer, CD133 expression is associated with treatment resistance, tumor metastasis, and recurrence [5, 6]. Hence, CD133 is a promising target for eradicating CSCs to improve advanced colorectal cancer management [7, 8].

For CD133-targeted strategies to succeed, it is necessary to be able to identify and quantify CD133 protein in tumors at baseline as well as monitor changes in expression during or after treatments. Immunohistochemical staining assessment of biopsied specimen is problematic due to the heterogeneity of CD133 expression according to the lesion site as well as within the same lesion [9]. Moreover, it does not allow quantitative measurement of the total protein amount in the whole tumor. Positron emission tomography (PET) imaging can be applied to overcome the limitations of biopsy-dependent techniques including sampling errors, invasiveness, and the challenges that are associated with conducting serial examinations. Indeed, immuno-PET using radiolabeled anti-CD133 antibodies has been associated with successful imaging of a tumor expressing CD133 [10–13].

Tsurumi et al. previously imaged CD133 on HCT116 colon carcinoma xenografts of mice using a specific AC133.1 monoclonal antibody that was fluorescently labeled [10]. Gaedicke et al. of this group later labeled the same antibody with ^64^Cu-NOTA to successfully visualize CD133-overexpressing subcutaneous and orthotopic glioma xenografts with PET [11]. Excellent tumor contrast was achieved in their study, even though PET was performed at 24 and 48 hours postinjection. However, longer lived ^89^Zr allows more delayed imaging of intact antibodies that often have long circulating times of up to several weeks [12]. Glumac et al. used ^89^Zr to radiolabel a different HA10 IgG for PET and confirmed greater uptake in CD133-positive compared to CD133-negative preclinical models of prostate cancer [13]. However, radiolabeling in their study was by random coupling to amine residues, which limits tracer homogeneity and target affinity of the antibody.

Importantly, tumor CD133 status has been recognized to be dynamic and with notable changes in response to the tumor microenvironment [14–16]. This underscores the need to better understand how tumor CD133 expression is regulated in living bodies. There is particularly rising interest in the role of proinflammatory mediators and signaling in the pathobiology and progression of colorectal cancer [17, 18]. The inflammatory microenvironment also has potential stimulatory effects on colon CSCs [19, 20]. Anti-inflammatory drugs are thus thought to favorably influence the biology of colorectal tumors. Celecoxib is a nonsteroidal anti-inflammatory drug (NSAID) that exerts antitumor effects through cyclooxygenase-2- (COX-2-) dependent [21] and independent pathways [21–23]. This has led to recent clinical trials to test the efficacy of celecoxib in combination with other drugs for treating colorectal cancer [24–26]. As such, the ability to monitor the effect of celecoxib on tumor CD133 expression could support the drug's capacity to suppress colon cancer stemness and provide rationale for its use to improve patient outcome. Whether immuno-PET is capable of providing quantitative measurements of changes in tumor CD133 status after treatment has not been previously explored.

In this study, we investigated the capacity of CD133-targeted ^89^Zr immuno-PET to noninvasively monitor celecoxib treatment-induced changes in endogenous CD133 protein expression on colon cancer cells and tumors. To maximize target affinity and specificity, we radiolabeled the specific monoclonal antibody AC133.1 by site-specific conjugation to cysteine residues. We further explored the signaling pathways involved in the observed effects of celecoxib on tumor CD133 expression and PET results.

## 2. Materials and Methods

### 2.1. Cell Culture and Reagents

HT29 and HCT116 human colon cancer cells and CT26 mouse colon cancer cells were from the American Type Culture Collection (ATCC), while HCT-15 and SNUC-5 human colon cancer cells were from the Korean Cell Line Bank (KCLB). All cell lines were maintained in 5% CO_2_ at 37°C in RPMI 1640 medium (Lonza, Basel, Switzerland) supplemented with 10% fetal bovine serum (FBS; Serena, Germany), 2 mM L-glutamine, and 100 U/ml penicillin-streptomycin (Gibco Laboratories, Gaithersburg, MD). Cells were subcultured twice a week and used when 80% confluence was reached.

Celecoxib was acquired from Sigma Chemicals (St. Louis, MO) and dissolved in dimethyl sulfoxide (DMSO). Rat IgG against mouse CD133 was purified from the supernatant of AC133.1 hybridoma cells (ATCC HB-12346), according to standard methods. A rabbit antibody against CD133 was acquired from ProteinTech Group Inc. (18470-I-AP; Rosemont, IL); rabbit antibodies against HIF-1*α* were acquired from Abnova (#PAB12138); rabbit antibodies against total (t-) and phosphorylated (p-) forms of AKT (#9272S and #9271S), I*κ*B*α* (#4812S and #2859S), and NF*κ*B (#8242S and #3033S) were acquired from Cell Signaling Technology (Danvers, MA); a mouse antibody against *β*-actin was acquired from Santa Cruz Biotechnology (#sc-47778; Santa Cruz, CA) and that against HDAC1 was from Cell Signaling (#5356S); horseradish peroxidase-conjugated secondary anti-rabbit and anti-mouse antibodies were acquired from Cell Signaling Technology.

### 2.2. Deferoxamine Conjugation and Site-Specific ^89^Zr Labeling of the Anti-CD133 Antibody

**Figure 1:**
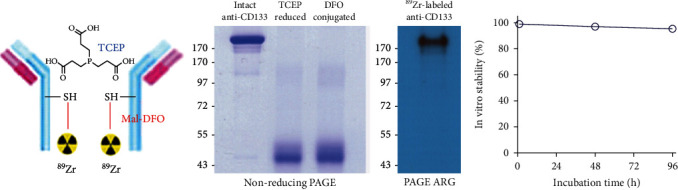
Deferoxamine conjugation and ^89^Zr labeling of anti-CD133 IgG. (a) Diagram of ^89^Zr-CD133 IgG (left), nonreduced SDS polyacrylamide gel electrophoresis (PAGE) of unmodified, TCEP-reduced, and DFO-conjugated anti-CD133 IgG (middle), and autoradiography of a native ^89^Zr-CD133 IgG PAGE (right). (b) In vitro stability of ^89^Zr-CD133 IgG assessed by radio-iTLC after incubation in PBS at 37°C for 96 hours (right).

Anti-CD133 IgG was site-specifically conjugated with deferoxamine- (DFO-) maleimide on sulfohydryl residues using previously described methods [27, 28] (Figure 1(a)). Briefly, 2 mg of antibody was incubated with 26.8 *μ*l (3.3 mM final concentration) of 100 mM tris(2-carboxyethyl)phosphine (TCEP; Sigma Chemicals) for 20 min at room temperature (RT) at a 1 : 100 molar ratio. Sulfohydryl residues of anti-CD133 IgG diluted in 0.1 M sodium phosphate that contained 150 mM NaCl and 1 mM EDTA were conjugated for 60 min at room temperature (RT) with 56.4 *μ*l of 2 mM N-(3,11,14,22,25,33-hexaoxo-4,10,15,21,26,32-hexaaza-10,21,32-trihydroxytetratriacontane)-maleimide (Macrocyclics, TX). The molar ratio of DFO-maleimide to antibody was 60 : 1. ^89^Zr-oxalate (50 *μ*l; Korea Atomic Energy Research Institute) was neutralized with 25 *μ*l of 2 M Na_2_CO_3_ and mixed with deferoxamine-conjugated anti-CD133 IgG in 75 *μ*l of 0.5 M HEPES buffer (pH 7.5). Following 60 min of incubation at RT with tapping every 15 min, the reaction mixture was eluted through a PD-10 column with 0.25 M sodium acetate containing 0.5% gentisic acid. Fractions of 0.5 ml were collected and counted for radioactivity on a high-energy *γ*-counter, and the peak activity fraction was used.

### 2.3. Polyacrylamide Gel Electrophoresis (PAGE) and Autoradiography

For nonreducing sodium dodecyl sulfate- (SDS-) PAGE, 2 *μ*g of intact, TCEP-reduced, and DFO-conjugated anti-CD133 IgG was diluted with water and 5x nonreducing sample buffer without dithiothreitol (DTT). Samples were boiled at 95°C for 10 min and then separated on an 8% sodium dodecyl sulfate (SDS) polyacrylamide gel by electrophoresis. The gel was subsequently stained with 0.5% Coomassie blue. Autoradiography was performed for ^89^Zr-CD133 IgG, which was separated by 8% native PAGE with sample buffer without SDS or DTT.

### 2.4. Radiochemical Stability Assessment

^89^Zr-CD133 IgG was tested for radiochemical purity and stability with radio-instant thin layer chromatography (radio-iTLC). The radiotracer was incubated in phosphate-buffered saline (PBS) at 37°C for indicated durations. Radio-iTLC was then performed using 50 mM ethylene diamine tetraacetic acid (EDTA, pH 5.5) as eluent on an iTLC-SG glass microfiber chromatography paper infused with silica gel (Agilent Technologies, CA). Under these conditions, an intact radiolabeled antibody remained at baseline while free ^89^Zr^4+^ ions and [^89^Zr]-EDTA migrated to the solvent front.

### 2.5. Cell Binding Assays

HT29 and HCT116 *c*ells were incubated with 74 kBq of ^89^Zr-CD133 IgG for 60 min at 37°C in RPMI 1640 medium. Cells were then washed twice with cold PBS (pH 7.4), lysed with 0.5 ml of 0.1 N NaOH, and measured for radioactivity on a high-energy *γ*-counter. Binding specificity was evaluated in the presence of 500 nM of cold anti-CD133 IgG.

### 2.6. Western Blotting of Cultured Cells and Tumor Tissue Protein

Total cellular protein was obtained in cultured cells with lysis buffer that contained proteinase and phosphatase inhibitor and in tumor tissues by homogenization. Briefly, cells were washed with cold PBS and solubilized at -20°C for 20 min with 200 *μ*l PRO-PREP protein extraction solution (iNtRON Biotechnology Inc., Korea) supplemented with protease inhibitor (P2714; Sigma) and phosphatase inhibitor cocktails (78420; Thermo Fisher Scientific). After centrifugation at 14,000 rpm and at 4°C for 10 min, the supernatant was stored at -70°C in a deep freezer until use.

To prepare the cytoplasmic and nuclear proteins, harvested cultured cells and homogenized tumor tissues were washed with cold PBS, spun down, and suspended in an ice-cold cytoplasmic extraction reagent-I from Thermo Scientific (Waltham, MA). After 10 min of incubation on ice, ice-cold cytoplasmic extraction reagent-II was added. The mixture was vortexed for 5 sec and incubated on ice for another 1 min. After centrifugation for 5 min at 16,000 × *g*, the supernatant was transferred to a clean prechilled tube and used as a cytoplasmic extract. The remaining pellet was washed with cold PBS and suspended in an ice-cold nuclear extraction reagent. The mixture was vortexed for 15 sec every 10 min for a total of four times and then centrifuged at 16,000 × *g* for 10 min. The supernatant was finally transferred to a prechilled tube and used as the nuclear extract. Samples were stored at -70°C until use.

Total cellular protein (20 *μ*g), cytoplasmic protein (20 *μ*g), and nuclear protein (10 *μ*g) were separated by 10% SDS-PAGE and transferred to polyvinylidene fluoride (PVDF) membranes. The membranes were incubated with primary antibodies at 4°C overnight. This included rabbit IgG against CD133 (1 : 5000), rabbit IgG against HIF-1*α* (1 : 2000), rabbit IgG against p-AKT (1 : 1000), rabbit IgG against p-NF*κ*B (1 : 1000), and rabbit IgG against p-I*κ*B*α* (1 : 1000). After washing with TBST buffer, membranes were incubated with HRP-conjugated secondary anti-rabbit IgG (1 : 10000 for CD133; 1 : 4000 for HIF-1*α*; 1 : 2000 for p-AKT, p-NF*κ*B, and p-I*κ*B*α*) at RT for 1 h. Immunoreactive protein was detected with enhanced chemiluminescence substrate, and band intensities were quantified. After visualizing the target protein, membranes were stripped and reincubated with mouse IgG against *β*-actin (1 : 5000), rabbit IgG against t-AKT (1 : 2000), rabbit IgG against t-NF*κ*B (1 : 2000), rabbit IgG against t-I*κ*B*α* (1 : 2000), or mouse IgG against HDAC1 (1 : 2000) as loading controls.

### 2.7. In Vivo Pharmacokinetics

All animal experiments were conducted in accordance with the National Institute of Health Guide for the Care and Use of Laboratory Animals and approved by the Samsung Biomedical Research Institute ethics committee. Pharmacokinetic analysis was performed in normal Balb/c mice intravenously injected with 1.85 MBq of ^89^Zr-CD133 IgG. Blood samples of 5 *μ*l were collected from the tail vein at predetermined intervals, measured for radioactivity on a high energy *γ*-counter, and expressed as % injected dose (%ID) per ml. Time activity curves of blood were fitted by nonlinear regression with GraphPad Prism 8.4.3 software (GraphPad Software Inc., San Diego, CA) using two-phase exponential decay equations. Early and late clearance rate constants (K1 and K2) and half-lives (T1/2*α* and T1/2*β*) were calculated as pharmacokinetic parameters.

### 2.8. Murine Tumor Model and Celecoxib Treatment

Tumor models were prepared in Balb/c-nu mice by subcutaneously injecting 5 × 10^6^ HT29 cells into the right shoulder. Experiments were performed when the tumors' diameter reached 0.5 cm at approximately 14 days after cell inoculation. Tumor-bearing mice underwent biodistribution and PET imaging with or without excess cold antibody blocking and after treatment with a vehicle or celecoxib. Five normal Balb/c mice were used for blood clearance analysis, three tumor-bearing Balb/c-nu mice per group were used for blocking experiments with PET and biodistribution, five tumor-bearing mice per group were used for celecoxib treatment experiments with PET and biodistribution, and five tumor-bearing mice per group were used for tumor CD133 expression and signaling pathway analysis.

### 2.9. In Vivo PET Imaging and Biodistribution Studies in Tumor Models

At 6 days after intravenous injection with 4.8 MBq of ^89^Zr-CD133 IgG, isoflurane-anesthetized tumor-bearing mice underwent PET/CT imaging on an Inveon scanner (Siemens Medical Solutions, Erlangen, Germany). Target specificity of the tumor uptake was assessed by a 1 h preinjection of a 5 : 1 molar ratio of a cold anti-CD133 antibody over the radiotracer.

Biodistribution studies were performed in HT29 tumor-bearing mice after PET/CT imaging. The mice were sacrificed by cervical dislocation, and major organs and tumors were extracted, weighed, and measured for radioactivity on a high energy *γ*-counter.

### 2.10. Celecoxib Treatment of Mouse Models

The effect of celecoxib treatment was investigated in HT29 tumor-bearing mice. For PET/CT imaging and biodistribution studies, randomly allocated groups of mice were intraperitoneally injected with 40 mg/kg celecoxib (*n* = 5) or DMSO in saline (control; *n* = 5) for every 2 days for a total of eight times. ^89^Zr-CD133 IgG was injected on the day of the seventh treatment. For Western blot analysis, separate groups of mice were intraperitoneally injected with vehicle (control; *n* = 6) or 40 mg/kg celecoxib (*n* = 6) every day for 4 consecutive days and then sacrificed the day after the final treatment.

### 2.11. Statistical Analysis

All data are presented as means ± SD. Significant differences between groups were analyzed by two-tailed unpaired Student's *t*-tests. *P* < 0.05 was statistically significant.

## 3. Results

### 3.1. DFO Conjugation and Site-Specific ^89^Zr Labeling of CD133 IgG

Site-specific conjugation of sulfohydryl residues on AC133.1 (anti-CD133 IgG) with DFO-maleimide was straightforward and efficient. Nonreduced SDS-PAGE demonstrated that treatment with TCEP completely reduced target disulfide bonds to produce half-sized antibody fragments, which remained reduced after conjugation with DFO-maleimide (Figure 1(a)). This supports site-specific reduction of the antibody at the two hinge region disulfide bonds by TCEP [29].

^89^Zr labeling of DFO-conjugated anti-CD133 IgG was reproducible with an efficiency of 65%. Autoradiography after PAGE of the first peak fraction of the column-elute displayed a clear radioactive band at the expected 170 kD region (Figure 1(a)). Radiochemical purity was 99%, and specific activity was 1.6 mCi/mg. Radiochemical stability assessed by iTLC analysis demonstrated >96% intact radiolabel after up to 96 h incubation in PBS (Figure 1(b)).

### 3.2. CD133 Expression in Colon Cancer Cells and ^89^Zr-CD133 IgG Binding

**Figure 2:**
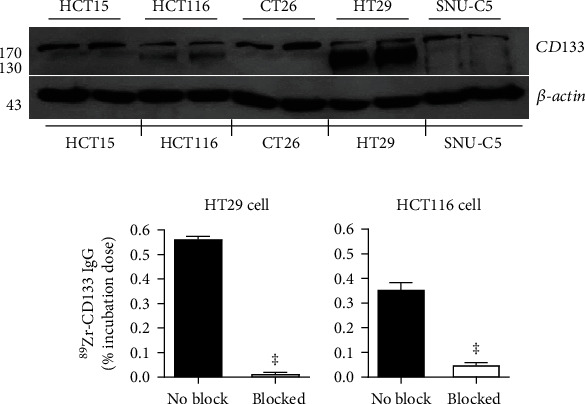
Colon cancer cell binding and pharmacokinetic properties of ^89^Zr-CD133 IgG. (a) Western blotting of CD133 protein in various colon cancer cell lines (top) and ^89^Zr-CD133 IgG binding to high-expressing HT29 and low-expressing HCT116 human colon cancer cells (bottom). (b) Specificity of binding was assessed by blocking with excess cold anti-CD133 IgG. Bars are means ± SD of % incubation dose obtained from a single representative experiment with triplicate samples. ^‡^*P* < 0.001.

Western blotting of protein from various colon cancer cell lines showed the highest CD133 expression in HT29 cells, moderate expression in CT26 and HCT15 cells, and lower expression in HCT116 and SNU-C5 cells (Figure 2(a)). Cell binding assays demonstrated that high CD133-expressing HT29 cells had significantly greater ^89^Zr-CD133 IgG binding compared to low-expressing HCT116 cells (0.56 ± 0.01 vs. 0.35 ± 0.03% incubated dose, *P* < 0.001; Figure 2(b)). The results also confirmed highly specific binding to both cancer cell types that were substantially inhibited to 2.8 ± 0.7% and 13.9 ± 3.1% of unblocked levels by 500 nM of cold anti-CD133 IgG, respectively (both *P* < 0.001; Figure 2(b)).

### 3.3. In Vivo Pharmacokinetic Properties of ^89^Zr-CD133 IgG

The pharmacokinetic profile of intravenously injected ^89^Zr-CD133 IgG in normal mice followed a biexponential pattern of blood clearance. Early K1 and late K2 rate constants of 0.11 and 0.02, respectively, led to an early distribution half-life (T1/2*α*) of 6.3 h and late clearance half-life (T1/2*β*) of 35.4 h, respectively (Figure 2(c)).

### 3.4. ^89^Zr-CD133 IgG PET and Biodistribution

**Figure 3:**
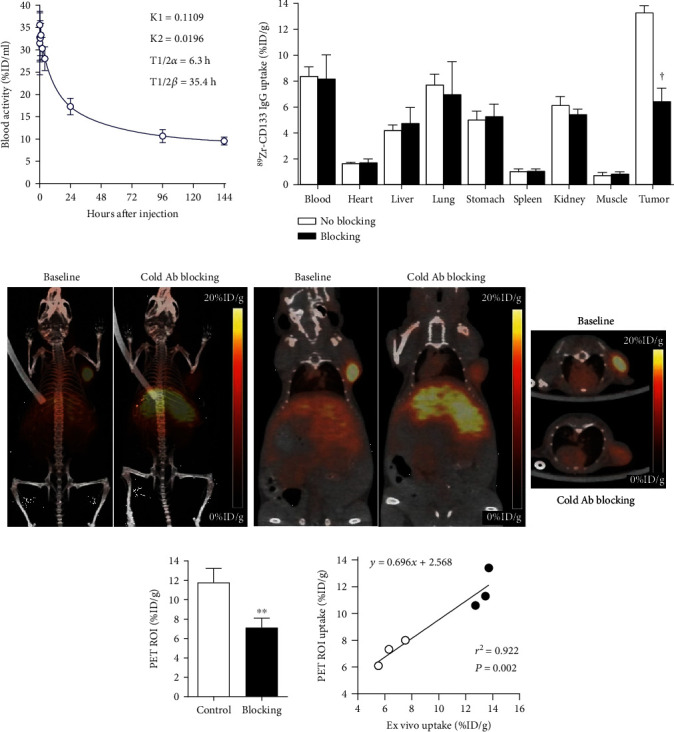
^89^Zr-CD133 IgG biodistribution and PET in mice. (a) Time-dependent blood clearance of ^89^Zr-CD133 IgG following intravenous injection in normal Balb/c mice (left) and 6-day biodistribution in HT29 tumor-bearing Balb/c nu mice with or without excess cold anti-CD133 IgG (right). The blood clearance curve was fitted with two-phase exponential decay nonlinear regression to obtain early and late rate constants (K1 and K2) and half-lives (T1/2*α* and T1/2*β*). (b) Representative maximal intensity projection (left) and coronal (middle) and transaxial (right) PET images at 6 days postinjection in tumor-bearing mice with or without excess cold IgG. (c) PET image-based tumor uptake level (left) and correlation between PET image-based and ex vivo count-based tumor uptake levels (right). All data are mean ± SD of %ID/g obtained from five (blood clearance) or three mice per group (biodistribution and PET). ^∗∗^*P* < 0.01, ^†^*P* < 0.005, compared to tumor uptake in the absence of excess cold IgG.

PET/CT imaging of mice at 6 days after ^89^Zr-CD133 IgG injection displayed clear HT29 tumor visualization (Figure 3(a)), along with rather low liver, spleen, and renal activities. PET showed substantially reduced tumor uptake in mice preinjected with excess cold anti-CD133 IgG.

Biodistribution analysis following PET imaging confirmed high ^89^Zr-CD133 IgG accumulation in HT29 tumors at 13.3 ± 0.5% injected dose per gram of tissue (%ID/g; Figure 3(b)). This was significantly higher than blood activity (8.4 ± 0.7%ID/g), as well as uptake by the liver (4.24 ± 0.4%ID/g), spleen (1.1 ± 0.2%ID/g), or kidneys (6.2 ± 0.7%ID/g; all *P* < 0.001). Preinjection of the excess antibody caused a substantial 51.4% reduction of tumor uptake, confirming specific targeting in vivo (Figure 3(b)). The cold antibody did not influence uptake in other organs.

### 3.5. Effects of Celecoxib Treatment on HT29 Colon Cancer Cells

**Figure 4:**
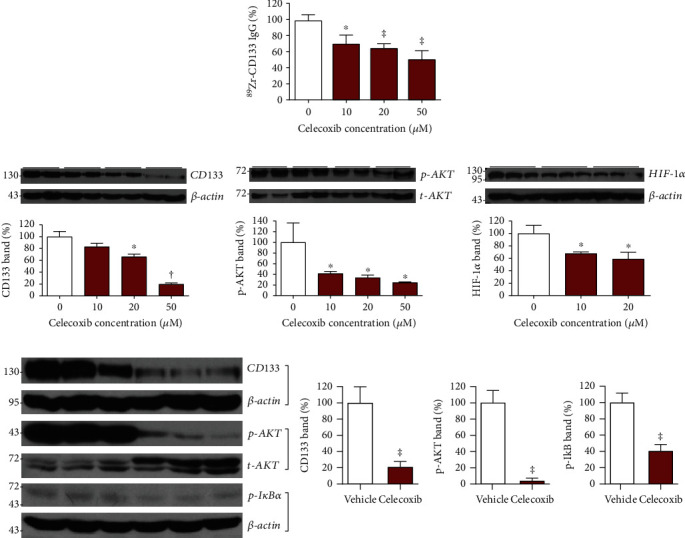
Effects of celecoxib on HT29 cell signaling pathways. (a) ^89^Zr-CD133 IgG binding. (b) Immunoblots and quantified band intensities of CD133 (left), HIF-1*α* (middle), and p-AKT (right) after 24 hours of treatment with graded doses of celecoxib. (c) Immunoblots and quantified band intensities of total cellular CD133 and p-AKT and cytosolic p-I*κ*B*α*. Bars are means ± SD of % controls obtained from triplicate (A binding and C) or duplicate samples per group (A immunoblots and B). Band intensities are corrected by *β*-actin (CD133 and p-I*κ*B*α*) or t-AKT (p-AKT). ^∗^*P* < 0.05, ^†^*P* < 0.005, and ^‡^*P* < 0.001, compared to controls.

We next investigated the effects of celecoxib treatment on cultured HT29 cells. The results showed that all celecoxib doses tested significantly reduced ^89^Zr-CD133 IgG binding and that 24 h treatment with a dose of 50 *μ*M reduced binding to 50.3 ± 10.9% of untreated controls (*P* < 0.001; Figure 4(a)). Immunoblotting revealed that this was accompanied by dose-dependent decreases in CD133 expression that were markedly suppressed to 19.9 ± 2.1% of controls by 50 *μ*M (*P* < 0.005; Figure 4(a)).

Investigation of candidate pathways revealed that celecoxib dose-dependently suppressed AKT activation, which led to p-AKT level that was reduced by a dose of 50 *μ*M to 24.7 ± 1.1% of levels noted in untreated controls (*P* < 0.05; Figure 4(b)). Celecoxib also modestly reduced HIF-1*α* level to 58.8 ± 10.9% of controls by a dose of 20 *μ*M (*P* < 0.05; Figure 4(b)). Repeat experiments by treatment with 50 *μ*M of celecoxib confirmed substantial reduction of CD133 levels to 20.8 ± 6.9% of controls and marked suppression of p-AKT levels to 4.2 ± 3.0% of controls (both *P* < 0.001; Figure 4(c)). The treatment modestly reduced p-I*κ*B*α* levels to 40.5 ± 7.9% of controls (*P* < 0.001; Figure 4(c)). Together, these results reveal that a near-complete blockade of AKT activation is associated with the celecoxib effect on downregulated CD133 expression and a weaker association of reduced I*κ*B*α* signaling.

### 3.6. Effects of Celecoxib on ^89^Zr-CD133 IgG PET and Tumor CD133 Expression In Vivo

**Figure 5:**
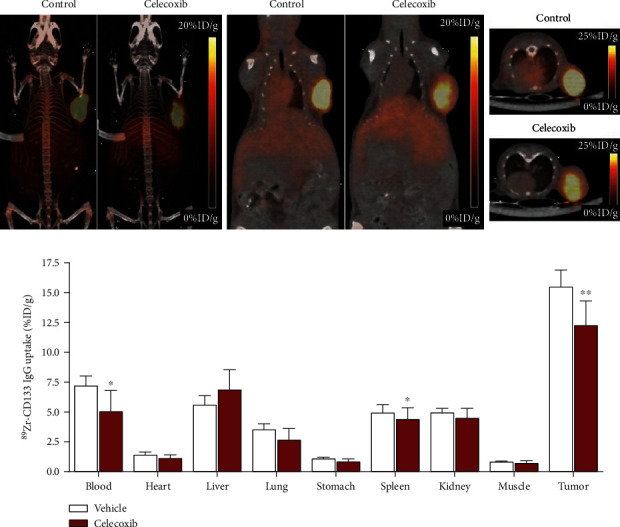
Effect of celecoxib on tumor uptake of ^89^Zr-CD133 IgG. (a) Representative maximal intensity projection (left) and coronal (middle) and transaxial (right) PET images in HT29 tumor-bearing mice following intraperitoneal injection of 40 mg/kg celecoxib or vehicle (control) every two days for a total of eight times. ^89^Zr-CD133 IgG was injected on the day of the seventh celecoxib treatment. (b) Biodistribution of ^89^Zr-CD133 IgG in tumor-bearing mice treated as above. Bars are mean ± SD of %ID/g obtained from five animals per group. ^∗^*P* < 0.05, ^∗∗^*P* < 0.01, compared to controls.

Immuno-PET/CT imaging of mice at 6 days displayed significantly reduced HT29 tumor uptake of ^89^Zr in animals treated with celecoxib compared to vehicle-administered controls (Figure 5(a)). The finding was confirmed by biodistribution studies that showed significantly decreased HT29 tumor uptake by celecoxib treatment to 12.3 ± 2.0%ID/g, compared to 15.5 ± 1.4%ID/g in vehicle-treated controls (*P* = 0.01; Figure 5(b)). Other organs did not show significantly different uptake levels, except for slight reductions in blood and spleen activity in celecoxib-treated mice compared to control animals.

**Figure 6:**
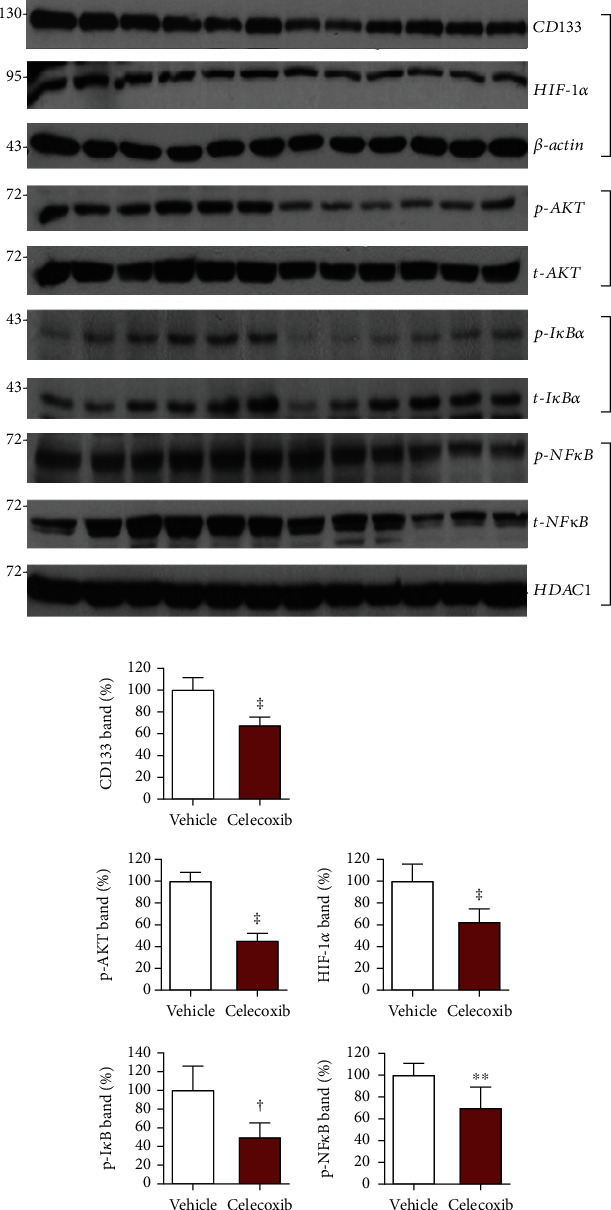
Effects of celecoxib on HT29 tumor expression of CD133 and signaling pathways. (a) Immunoblots of cellular CD133, p-AKT, HIF-1*α*, and p-I*κ*B*α* and nuclear p-NF*κ*B. Tumor-bearing mice were intraperitoneally injected daily with vehicle or celecoxib for four consecutive days and were sacrificed on the day of the final treatment. (b) Quantified band intensities of CD133 and HIF-1*α* corrected by *β*-actin, p-AKT corrected by t-AKT, p-I*κ*B*α* corrected by t-I*κ*B*α*, and p-NF*κ*B corrected by HDAC1. Bars are mean ± SD of %ID/g obtained from six animals per group. ^∗∗^*P* < 0.01, ^†^*P* < 0.005, and ^‡^*P* < 0.001.

Immunoblotting demonstrated weaker CD133 protein bands in HT29 tumor tissue of mice treated with celecoxib (67.5 ± 7.8% of controls) compared to stronger CD133 protein bands in tumor tissue of vehicle-treated controls (*P* < 0.005; Figures 6(a) and 6(b)). Assessment of the potential signaling pathways indicated that tumors of celecoxib-treated mice showed substantially reduced p-AKT levels (45.0 ± 7.1%) and modestly reduced HIF-1*α* levels (62.3 ± 12.3% of vehicle-treated control; both *P* < 0.001; Figures 6(a) and 6(b)). Tumors of celecoxib-treated mice also showed that p-I*κ*B*α* reduced to 49.5 ± 15.7% (*P* < 0.005) and p-NF*κ*B reduced to 69.6 ± 19.3% of vehicle-treated controls (*P* < 0.01; Figures 6(a) and 6(b)).

## 4. Discussion

In this study, we developed an immuno-PET technique that targets CD133 expression and noninvasively monitors its modulation in colon tumors of living mice.

We used a rat monoclonal anti-CD133 IgG1 produced by AC133.1 hybridoma cells that is widely applied for colon CSC isolation and characterization. This antibody was previously used to investigate CD133 expression and function by Western blotting [30], immunochemistry [13, 31], and FACS analysis [13, 30, 31]. Target specificity of the antibody was confirmed based on binding to CD133-positive but not CD133-negative tumor cells [10]. This antibody was also previously used for in vivo optical imaging of CD133 on colon carcinoma xenografts following fluorescence labeling [10]. Furthermore, radiolabeling of this antibody with ^64^Cu-NOTA provided excellent PET images of CD133-overexpressing glioma xenografts at 48 hours [11]. The impressive early tumor contrast obtained in this study is likely contributed by strong tumor cell CD133 expression by lentiviral transduction. This is because large quantities of target expression not only enhance tumor binding but also facilitate blood clearance by the tumor acting as a sink organ. Under usual conditions, intact antibodies show long circulating half-lives that are reported to exceed 25 days [12]. Therefore, longer intervals are generally required for immune-PET of endogenous protein expression, and radiolabeling with ^89^Zr is beneficial in this instance.

Glumac et al. labeled ^89^Zr to a different HA10 IgG and successfully imaged CD133-positive tumors of preclinical prostate cancer models [13]. However, ^89^Zr labeling was by random coupling to amine residues. Although tumor uptake at 72-hour postinjection in this study reached greater levels than that of our results at 6 days, this was again in tumor cells transduced with lentivirus for CD133 overexpression. In our study, we sought to investigate the capacity of immuno-PET to monitor changes in endogenous CD133 expression, which is relatively scarce compared to the remarkably high levels in cells transduced for overexpression. Therefore, a more delayed time point was required for sufficient tumor binding and blood clearance. The 6-day time point used in our study is within the 4 to 8-day range reported to be generally required for optimal tumor contrast in ^89^Zr immune-PET of murine models [32]. Clinical immuno-PET in human subjects is also typically performed at 5–7 days postinjection of ^89^Zr-labeled antibodies [33].

Cysteine-specific conjugation using DFO-maleimide is an elegant way of tailoring the site for ^89^Zr attachment to antibodies for better radioprobe homogeneity and immunoreactivity [27, 28]. In our study, ^89^Zr-CD133 IgG prepared by this technique showed high colon cancer cell binding that was markedly suppressed by excess cold antibody, confirming good immunoreactivity and target specificity. SDS-PAGE analysis of TCEP-treated antibodies indicated reduction and DFO conjugation at the two hinge region disulfide bonds, consistent with the preferential reduction of these regions to yield monovalent components with free thiol groups for site-directed radiolabeling [29].

Biodistribution and PET studies were performed in mice bearing tumors from HT29 cancer cells, which are useful for studying CD133-positive colon CSCs [34, 35]. ^89^Zr-CD133 IgG PET of the mice displayed high tumor uptake with excellent contrast that was reduced by 20.6% when the cold antibody was coinjected, confirming CD133-specific tumor targeting. There were relatively lower blood pool activity and lower uptake in normal organs, which is an important advantage for imaging of intra-abdominal tumors including those of the colon. Therefore, ^89^Zr-CD133 IgG was confirmed to have excellent target specificity and favorable in vivo biodistribution and to provide high-contrast tumor PET imaging.

Adjuvant chemotherapy with optimal regimens is important to improve survival of patients with regional lymph node-positive colon cancer because they frequently develop disease recurrence when treated with surgery alone. However, significant variability remains for individual outcomes in advanced colon cancer, and additional strategies are needed to maximize the treatment efficacy [24]. Nonsteroidal anti-inflammatory drugs and selective COX-2 inhibitors have been recognized as agents that may influence colon cancer progression [36], and observational studies have shown that usage of COX-2 inhibitors in colorectal cancer lowers risk of recurrence [37]. In the present study, celecoxib treatment was found to significantly suppress CD133 expression in HT29 colon cancer cells and HT29 tumors. Similar observations have been made in renal cell carcinoma cells [38], pancreatic cancer cells [39], and colon cancer cells [40]. In murine tumor models, celecoxib reduced colon cancer metastasis and number of CSCs [19]. Moreover, clinical studies have indicated that preoperative treatment of patients with NSAIDs can downregulate colon cancer tissue expression of CD133 [41, 42].

Together, these findings indicate that the use of COX-2 inhibitors could help suppress a colorectal tumor expressing CD133, a major cancer stemness biomarker. The success of this strategy would clearly benefit from the ability to select the best drug or drug combination and dosing schedule for this purpose. Based on our results, ^89^Zr-CD133 IgG provided information on celecoxib-induced downregulation of the colon cancer CD133 protein by displaying significantly decreased cell binding in vitro and reduced tumor uptake in vivo. These results support an opportunity for chemotherapeutic elimination of colon cancer cells with stemness properties and a role for ^89^Zr-CD133 IgG to noninvasively monitor this response in vivo.

Mechanistically, the tumor-suppressive actions of celecoxib have been associated with a direct influence on several intracellular signaling molecules in addition to COX-2 inhibition [22, 23, 43]. Certain oncogenic factors that drive tumor growth have been suggested to stimulate CD133 transcription, and the ability of celecoxib to downregulate colon cancer CD133 might occur by inhibiting such oncogenic signaling molecules [40]. In our study, the effect of celecoxib on CD133 expression in HT29 cells and tumors was accompanied by marked suppression of AKT activation. Reductions in HIF-1*α* accumulation and I*κ*B*α*/NF*κ*B phosphorylation were also seen, indicating downregulation of hypoxic and inflammatory signaling. It has been previously shown that CD133 overexpression can activate AKT [44]. Conversely, celecoxib was observed to be capable of inhibiting AKT through PTEN upregulation [45], and celecoxib in combination with radiotherapy was shown to downregulate AKT signaling [23]. Our results thus support the role of AKT inhibition in the ability of celecoxib to suppress colon cancer CD133 expression. This not only highlights a major mechanism for the modulation of tumor CD133 expression but also suggests the potential benefit of combining AKT inhibitors to improve colorectal cancer therapy.

Based on our findings, ^89^Zr-CD133 IgG PET could be used for screening drugs for the capacity to suppress colon cancer stemness by noninvasively comparing tumor CD133 status before and after chemotherapy. Moreover, ^89^Zr-CD133 IgG PET may be helpful for selecting the optimal combination treatment protocol, including timing, dosage, and sequence of administration to optimize best patient outcomes.

## 5. Conclusion

^89^Zr-CD133 IgG showed target-specific binding with favorable in vivo pharmacokinetics and provided high-contrast tumor PET imaging. Celecoxib treatment suppressed CD133 expression in HT29 cells and tumors, and this was faithfully represented by reduced ^89^Zr-CD133 IgG binding and tumor uptake. Mechanistically, these responses were found to occur through AKT inhibition. Thus, ^89^Zr-CD133 IgG PET may be useful for screening drugs that can help eliminate colon cancer cells with stemness properties.

## Data Availability

All data generated or analyzed during this study are included in this published article.

## Additional Points

*Key Points*. (i) Question: can immuno-PET based on a site-specific ^89^Zr-labeled anti-CD133 antibody be used to noninvasively monitor changes in tumor CD133 expression induced by chemotherapy? (ii) Relevant findings: ^89^Zr-CD133 IgG showed CD133-dependent specific binding to HT29 colon cancer cells, displayed favorable in vivo pharmacokinetics and biodistribution, and provided high-contrast PET imaging of HT29 tumors. Celecoxib significantly suppressed HT29 cell CD133 expression through AKT inhibition, and this was faithfully represented by reduced cellular ^89^Zr-CD133 IgG binding in vitro and tumor ^89^Zr-CD133 IgG uptake in vivo. (iii) Implications for patient care: ^89^Zr-CD133 IgG PET may be useful for noninvasively monitoring CD133 modulation to screen for drugs that can suppress cancer stemness properties.
